# Transmission Line Vibration Damper Detection Using Deep Neural Networks Based on UAV Remote Sensing Image

**DOI:** 10.3390/s22051892

**Published:** 2022-02-28

**Authors:** Wenxiang Chen, Yingna Li, Zhengang Zhao

**Affiliations:** 1 Faculty of Information Engineering and Automation, Kunming University of Science and Technology, Kunming 650500, China; wenxiang.chen@stu.kust.edu.cn (W.C.); zhaozhengang@stu.kust.edu.cn (Z.Z.); 2 Computer Technology Application Key Lab of the Yunnan Province, Kunming 650500, China

**Keywords:** power transmission lines, vibration dampers detection, unmanned aerial vehicle (UAV), deep neural networks, attention mechanism

## Abstract

Vibration dampers can greatly eliminate the galloping phenomenon of overhead transmission wires caused by wind. The detection of vibration dampers based on visual technology is an important issue. The current vibration damper detection work is mainly carried out manually. In view of the above situation, this article proposes a vibration damper detection model named DamperYOLO based on the one-stage framework in object detection. DamperYOLO first uses a Canny operator to smooth the overexposed points of the input image and extract edge features, then selectees ResNet101 as the backbone of the framework to improve the detection speed, and finally injects edge features into backbone through an attention mechanism. At the same time, an FPN-based feature fusion network is used to provide feature maps of multiple resolutions. In addition, we built a vibration damper detection dataset named DamperDetSet based on UAV cruise images. Multiple sets of experiments on self-built DamperDetSet dataset prove that our model reaches state-of-the-art level in terms of accuracy and test speed and meets the standard of real-time output of high-accuracy test results.

## 1. Introduction

The main function of a power line vibration damper is to reduce the vibration of the wire caused by wind galloping. High-voltage transmission towers have large spacing, which makes it easy for the wires to vibrate when subjected to wind. The periodic bending of the suspension caused by the vibration of the wire leads to fatigue damage to the metal wire. In severe cases, accidents such as wire breakage and power tower collapse will be induced. The use of a vibration damper on high voltage transmission lines can reduce the vibration of the wires caused by the wind, thereby reducing the probability of accidents. Therefore, vibration damper detection is an important topic in the inspection of overhead transmission lines [1]. Vibration damper detection refers to obtaining the specific position of the vibration damper in the inspection image. This task is an important prerequisite for the work of vibration damper displacement detection, damage detection, and corrosion detection. At present, vibration damper detection has attracted the attention of researchers in the fields of smart grid and machine vision, with certain progress made [2].

UAV technology has developed rapidly in recent years. UAV has the advantages of convenient operation, easy portability, and low cost [3]. Multi-UAV systems based on wireless sensor networks [4] are used in crop yield estimation [5], object detection [6], and other fields. UAVs have rapidly developed into important auxiliary equipment.

At present, the inspection of overhead transmission lines still mainly relies on visual inspection by staff, which can produce omissions and incorrect judgments for the vibration damper located at a high place; therefore, the use of UAVs for transmission line inspection is an issue of great research value. Researchers have used UAVs for equipment detection and other tasks [7,8]. This article focuses on the issue of vibration damper detection using UAV aerial images.

In early research work, traditional image processing techniques were most widely used in power line inspection scenarios [2,9]. Researchers would select appropriate feature extraction operators according to the actual situation and complete the task of object detection through a threshold setting. Machine learning algorithms were also selected to achieve better detection results [10]. However, such methods are very susceptible to interference from background information, especially when using UAV aerial photography data, as the similar color properties of vibration dampers and power towers can easily cause missed detection.

In recent years, with the exponential growth of machine computing power and data volume, it has become a research hotspot again. Deep learning technologies, especially convolutional neural networks, have opened new research directions in the field of computer vision. There is much research on power components using the state-of-the-art method in the field of object detection [11,12]. However, at present, these works are mainly based on the simple application of the framework, there is no targeted improvement for the characteristics of the vibration damper, and high accuracy of the model requires a large amount of computing resources.

In addition, some studies have used special equipment for imaging or for the physical properties of the device [13,14]. The results of these works are usually excellent, but the extra equipment overhead and high usage cost make such methods unsuitable for power line patrol scenarios.

Aiming at the research status of image-based vibration damper detection, this article proposes a vibration damper detection model based on the one-stage algorithm in target detection. The main contributions of this paper are as follows:

- A proposed vibration damper detection model called DamperYOLO based on the YOLOv4 framework, which is more robust than traditional methods and can achieve a good balance between speed and accuracy, and a vibration damper detection dataset called DamperDetSet based on UAVs aerial images.
- To enhance images, Gaussian filtering is used to smooth the overexposed points in the aerial image and the Canny algorithm is used to extract the contour information in the image.
- Introduction of an attention-based structure in the backbone of DamperYOLO. This module can introduce the edge information extracted by Canny into the forward propagation process of the model and provide semantic guidance for the feature extraction of the network.
- Addressing the problem that the vibration damper is small and difficult to detect in the UAVs aerial image, we used a feature fusion network based on FPN after the backbone. While outputting feature maps of different resolutions, the semantics and underlying feature information of each layer are maintained, which provides a high-quality data basis for the identification of vibration dampers.

The remainder of this article is organized as follows. Section 2 briefly introduces the related work of vibration damper detection. Section 3 introduces the basic framework used in the method proposed in this article. In Section 4, this article introduces the details of DamperYOLO. In Section 5, this article introduces the damper dataset, the experimental details, and a series of comparative experiments. Section 6 provides a brief summary of the work.

## 2. Related Work

This section focuses on the image-based vibration damper detection research. The existing work is mainly divided into traditional image processing methods, deep learning-based research, and detection methods based on auxiliary equipment.

### 2.1. Traditional Method

Traditional image processing algorithms use edge detection, color space conversion, and clustering algorithms to extract damper information in images, usually combined with machine learning algorithms for iterative classification tasks.

Wu et al. [2] used the snake model to extract the edge of the vibration damper, but due to the helicopter airborne imaging equipment required, the cruise cost was high. Huang et al. [9] performed corrosion and displacement detection on the vibration damper based on rusty area ratio and color shade index, involving grayscale processing, edge detection, threshold segmentation, morphological processing, and other technologies. Similarly, Song et al. [15] detected the rust problem of the vibration damper based on the histogram. Jin et al. [10] used Harr-like features and a cascade adaboost classifier to classify and detect vibration dampers on overhead lines. Yang et al. [16] performed exponential transformation on the S and V components in the HSV color space to improve the contrast between the front and background. Liu et al. [17] used the canny operator and Hough transform method to detect the displacement of the vibration damper on the high-voltage line. Similarly, Chen et al. [18] used random Hough transformation for the vibration damper detection task. Miao et al. [19] used the wavelet modules maximum method to locate the shock hammer on the transmission line. Pan et al. [20] used a simple extraction operator to monitor the state of the vibration damper. Jin et al. [21] used the Adaboost algorithm to conduct real-time monitoring of the line vibration damper through drones.

Traditional methods use operators and classifiers to identify the vibration damper on the line; the detection accuracy is limited by the complexity of the environmental background, but its advantage lies in its fast detection speed, which is suitable for real-time detection.

### 2.2. Deep Neural Networks

With the rapid development of deep learning technology, the detection of power line components based on neural networks such as CNNs has gradually become a popular research direction.

Based on YOLOv4, Bao et al. [1] used k-means to analyze the aspect ratio of the anchor to detect damage, corrosion, and displacement faults of the vibration damper. Zhang et al. [11] also used Faster R-CNN to detect damage and corrosion defects of the vibration damper twice, in which the first detection result was used as the second proposal, thereby improving the detection effect. Bao et al. [12] used the Cascade R-CNN framework to locate and detect the damage of the vibration damper. Yang et al. [16] performed the detection task of vibration dampers using Faster R-CNN based on HSV color space transformed images. Guo et al. [22] used YOLOv4 to improve the detection effect of damaged vibration damper. Wang et al. [23] investigated insulator defects in overhead transmission lines, damage to vibration dampers, and foreign objects in bird’s nests. Zhang et al. [24] switched to VGG16 as the basic backbone network and performed detection tasks for shockproof hammers and other foreign objects on power towers.

The detection of power line components and foreign objects using deep neural networks has also attracted the attention of researchers. For example, the YOLO framework is used to detect insulators on transmission lines [25] and icing detection [26], change the anchor setting of Faster R-CNN according to the shape characteristics of the insulator [27], using Mask R-CNN to detect line foreign objects [28], defect detection for high-speed rail catenary insulators [29], and detection of wet insulators using infrared images [30]. Usually, these studies are only simple applications of power components datasets, and most of the studies lack targeted transformation for specific environments and scenarios; the solutions provided are mostly trick stacking.

### 2.3. Auxiliary Equipment

In addition to using common optical images, there is also research that uses other imaging equipment and auxiliary devices to perform detection tasks. For example, a robot is used to reset the vibration damper [14,17,31], and the damage of the vibration damper is detected based on LiDAR data [13]. The damping of the vibration damper is detected based on sensors such as optical ground wire (OPGW) and an all-dielectric self-supporting (ADSS) optical cable [32]. In addition, some researchers [33] designed a rotation-free spacer damper to improve the anti-galloping ability of power lines.

### 2.4. Researches Summary

There is still room for improvement in the detection of vibration dampers for overhead transmission lines. A summary of these research is as follows:

- Traditional methods based on image processing technology. The detection accuracy is mostly dependent on the quality of the image. If the background in the image is too complex, this leads to the problem that the used feature operator does not cover all situations, which inevitably leads to a decrease in the detection accuracy. The advantage of the traditional method is that it consumes less resources and the calculation speed is fast. Therefore, at present, this type of method is still the most important when the scene is relatively simple, background interference is low, and the real-time requirement is high.
- The method based on deep neural network is the hottest research direction in the field of vibration damper detection. By relying on powerful computing equipment and a large amount of training data, an end-to-end network model can be obtained; on this basis, it is very easy to carry out detection tasks. However, there is currently no public dataset for the vibration damper of overhead transmission lines, and the detection effect of the model is often limited by the lack of computing power of edge devices.
- There is some research work based on auxiliary equipment. Such research uses the characteristics of ultrasonic or infrared imaging equipment to perform the task of vibration damper breakage detection. However, these devices are often inconvenient for use along complex overhead lines, and the maintenance and use costs of the devices are much higher than those of drones.

Combining the characteristics of the abovementioned research work, we not only hope to obtain excellent detection results, but also hope that the model can run in real time on devices lacking computing resources, such as drones. A one-stage method using deep neural network is the most suitable choice. One-stage object detection utilizes the powerful feature extraction capabilities of CNNs to cope with complex application scenarios. At the same time, the detection result does not depend on the proposal, and its calculation speed is fast enough. Therefore, in the following work, based on the one-stage model, we propose a detection method based on the visual characteristics of the vibration damper in the real scene.

## 3. Basic Knowledge of YOLO

YOLO [34] proposed by Redmon et al. in 2016 is a classic one-stage object detection method. YOLOs [34,35,36,37] solves the target detection problem as a regression problem. After an inference of the input image, the positions of all objects in the image, their categories, and corresponding confidence probabilities can be obtained. YOLO divides the input image into SxS grids, and each grid is responsible for detecting objects that fall into the grid. If the coordinates of the center position of an object fall into a grid, then the grid is responsible for detecting the object.

The difference between the backbone in YOLOv4 [37] is that it is based on the Darknet structure in YOLOv3 [36] and borrows the structure of the CSPNet network [38] to propose a network structure called CSPDarknet. The loss function used in training is CIOU [39].

Since the objects to be detected in this paper are only vibration dampers, an overly complex network structure will have a negative impact on feature extraction; therefore, this paper selects the classic ResNet101 [40] as feature extraction network. The objective function of YOLOv4 is as follows:

(1) $$L_{det}=L_{box}+L_{obj}+L_{cls}$$

where $L_{box}$, $L_{obj}$, and $L_{cls}$ represent the regression loss, confidence loss, and category loss of the box, respectively. The expression of the box regression loss is as follows:

(2) $$L_{box}=\lambda_{coord}\sum_{i=0}^{S^{2}}\sum_{j=0}^{B}1_{i,j}^{obj}\left(1-\left(IoU-\frac{Distance\_2^{2}}{Distance\_C^{2}}-\frac{v^{2}}{\left(1-IoU\right)+v}\right)\right)$$

where $\lambda_{coord}$ is the weight of box regression loss, $S_{i}^{2}$ represents the *i*th grid of *S*×*S* size, $B_{j}$ represents the *j*th predicted box of $S_{i}^{2}$, and $1_{i,j}^{obj}$ indicates that there is a target center of the prediction category in the box. IoU is the Intersection-of-Union of the predicted box and ground truth, the calculation formula of IoU is Equation (3), Distance_2 is the Euclidean distance between the center coordinates of $Box^{p}$ and $Box^{gt}$, Distance_C is the diagonal length of the smallest bounding rectangle of $Box^{p}$ and $Box^{gt}$, v is a parameter to measure the consistency of the aspect ratio of $Box^{p}$ and $Box^{gt}$, and the calculation formula of v is Equation (4).

(3) $$IoU=\frac{\left|Box^{p}\cap Box^{gt}\right|}{\left|Box^{p}\cup Box^{gt}\right|}$$

where $Box^{p}$ and $Box^{gt}$ represent the predicted box and ground truth, respectively.

(4) $$v=\frac{4}{\pi^{2}}{\left(arctan\frac{w^{gt}}{h^{gt}}-arctan\frac{w^{p}}{h^{p}}\right)}^{2}$$

where $w^{gt}$ and $w^{p}$ represent the width of the ground truth and predicted box, respectively, while $h^{gt}$ and $h^{p}$ represent their respective heights.

Similar to the regression loss, the loss function for the target prediction confidence is as follows:

(5) $$L_{obj}=\lambda_{noobj}\sum_{i=0}^{S^{2}}\sum_{j=0}^{B}1_{i,j}^{noobj}{\left(c_{i}-{\hat{c}}_{i}\right)}^{2}+\lambda_{obj}\sum_{i=0}^{S^{2}}\sum_{j=0}^{B}1_{i,j}^{obj}{\left(c_{i}-{\hat{c}}_{i}\right)}^{2}$$

where $\lambda_{noobj}$ and $\lambda_{obj}$, respectively, represent the weight of the confidence loss when the object is not included and when it is included. $c_{i}$ and ${\hat{c}}_{i}$, respectively, represent the true value and predicted value of whether there is an object of category *i* in the current box. The other parameters have the same meaning as in the regression loss.

The category prediction loss uses the classic cross-entropy loss, and its calculation formula is as follows:

(6) $$L_{obj}=\lambda_{noobj}\sum_{i=0}^{S^{2}}\sum_{j=0}^{B}1_{i,j}^{noobj}{\left(c_{i}-{\hat{c}}_{i}\right)}^{2}+\lambda_{obj}\sum_{i=0}^{S^{2}}\sum_{j=0}^{B}1_{i,j}^{obj}{\left(c_{i}-{\hat{c}}_{i}\right)}^{2}$$

where $\lambda_{class}$ represents the weight of the category loss; ${\hat{p}}_{i}(c)$ represents the predicted value of the confidence of the current category; and $p_{i}(c)$ is a conditional probability, which is obtained by obtaining a value of 0 or 1, depending on whether $S_{i}^{2}$ contains the target center, and then multiplying it with IoU.

YOLOv4 uses CSPDarknet53 [38] as its feature extraction network, but CSPDarknet53 has lots of parameters. In addition, the only object to be detected in this paper is the damper. As shown in Table 1, ResNet101 is composed of multiple groups of residual blocks. ResNet has excellent feature extraction ability, which overcomes the problem of low learning efficiency caused by excessive network depth. Therefore, the classic ResNet101 is used as the backbone in this article.

## 4. DamperYOLO

In this section, a new framework named DamperYOLO is proposed for the vibration damper detection task of overhead transmission lines based on YOLOv4 [37], Canny algorithm [41], attention mechanism [42] and FPN [43] structure.

### 4.1. Edge Extraction

The quality of the input image is very important as it is the first step of the whole network detection, which directly affects the subsequent detection process. Although, strong noise immunity is one of the advantages of deep neural networks, no network would want to receive a high-quality input, so that the trained model parameters have more powerful attention to our target. Therefore, we decided to use edge detection techniques to improve the semantic information in images for the purpose of image enhancement, detailed in this subsection.

The canny algorithm is used to extract edge information from UAV aerial images. The canny algorithm is mainly divided into four parts: Gaussian smooth image, gradient magnitude and direction calculation, gradient magnitude nonmaximum suppression, double threshold algorithm detection and edge connection.

Our images are obtained by unmanned aerial photography and are highly susceptible to light reflections to generate exposure points. To reduce the influence of these bright white points, a Gaussian kernel is used to smooth the image.

Compared with the median filter [44] and the mean filter [45], the Gaussian filter assigns different calculation weights to different fields of the current element, which can achieve the purpose of denoising while preserving the gray distribution characteristics of the image. Gaussian filtering is usually implemented by iterative operations on the image with (2*k* + 1) × (2*k* + 1) convolution kernels. The kernel generation equation is shown in Equation (7).

(7) $$H_{ij}=\frac{1}{2\pi \sigma^{2}}\exp\left(-\frac{{\left(i-\left(k+1\right)\right)}^{2}+{\left(j-\left(k+1\right)\right)}^{2}}{2\sigma^{2}}\right);\;1\leq i,j\leq \left(2k+1\right)$$

where *k* represents an integer, (2*k* + 1) represents the size of the convolution kernel, and (*i*, *j*) represents the coordinates of one of the points.

The size of the convolution kernel is usually set to an odd number for the convenience of calculation. The larger the kernel, the stronger the processing ability for local noise. In our experiments, kernels with sizes of 3 × 3, 5 × 5, and 9 × 9 were selected for comparison. The experimental results show that the kernel of 5 × 5 has the smallest effect.

After Gaussian smoothing, the background part still contains overexposed points. There is no need to worry about the negative impact this brings to the model, as the network focuses on the ground truth part during training. What must pay attention to is if the feature of the vibration damper is improved, and edge detection is one of the important means of image enhancement. The parts of the image with high gradient variation in the canny algorithm task image represent a higher probability of edges. Therefore, our next step is to extract the gradient information of the image.

Gradients reflect the intensity of local pixel transformations. The greater the gradient change, the greater the change in the corresponding region. The gradient needs to calculate the direction and size of two parts, usually by calculating the gradient of the horizontal and vertical directions to represent a complete gradient. Its calculation formula is shown in Equations (8) and (9).

(8) $$\frac{\partial f}{\partial x}\approx \frac{f(x+1,y)-f(x-1,y)}{2}$$

(9) $$\frac{\partial f}{\partial y}\approx \frac{f(x,y+1)-f(x,y-1)}{2}$$

The direction a and increment b of the gradient can be obtained based on the gradients in the horizontal and vertical directions, as shown in Equations (10) and (11).

(10) $$\theta =\tan^{-1}(\frac{\partial f}{\partial y}/\frac{\partial f}{\partial x})$$

(11) $$\left\|\nabla f\right\|=\sqrt{{\left(\frac{\partial f}{\partial x}\right)}^{2}+{\left(\frac{\partial f}{\partial y}\right)}^{2}}$$

Gradient images contain all grayscale variations. Therefore, the canny algorithm uses the nonmaximum suppression method [41] to propose the lower gradient variation in the region.

The nonmaximum suppression algorithm calculates in eight areas around the pixel, retaining the parts with the largest grayscale changes in the horizontal, vertical, and diagonal directions while eliminating other parts with smaller changes by changing the broad-side gradient map to a single pixel width of the side.

The method of the nonmaximum suppression algorithm can only enhance the edge information and cannot guarantee that the remaining part is foreground information. Therefore, the last step of the canny algorithm is to use the double threshold algorithm to separate the foreground and background based on our prior knowledge.

In the double-threshold algorithm, the pixels above the strong edge threshold represent edge information, and the pixels below the weak edge threshold represent background information. The threshold between the two is the pending element, and if there is a strong edge in the eight-neighborhood of these pixels, the pixel is also classified as an edge pixel. Through comparison experiments of 200, 300, and 400 strong edge thresholds, it was found that the threshold of strong edge is best when the threshold is 300, and the weak edge threshold is set to 0.5 times of the strong edge. The formula for classifying gradient map pixels is shown in Equation (12).

(12) $$f\left(i\right)=\left\{\begin{array}{l} \mathrm{strong}\;\mathrm{edge}\;;\;\;\;i>300\\ \mathrm{weak}\;\mathrm{edge}\;;\;\;150\leq i\leq 300\\ \mathrm{non}-\mathrm{edge}\;;\;\;\;\;i<150 \end{array}\right.$$

**Figure 1:**
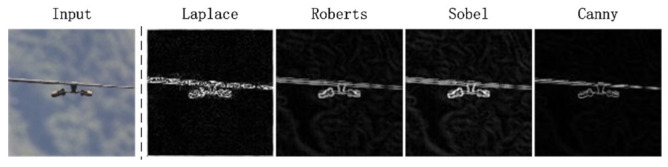
Test examples of edge detection algorithm.

To verify the effect of edge detection, we compared the performance of several classical edge detection operators on vibration dampers. As shown in Figure 1, the edge extracted by the Canny operator is the clearest.

### 4.2. Attention Mechanism

**Figure 2:**
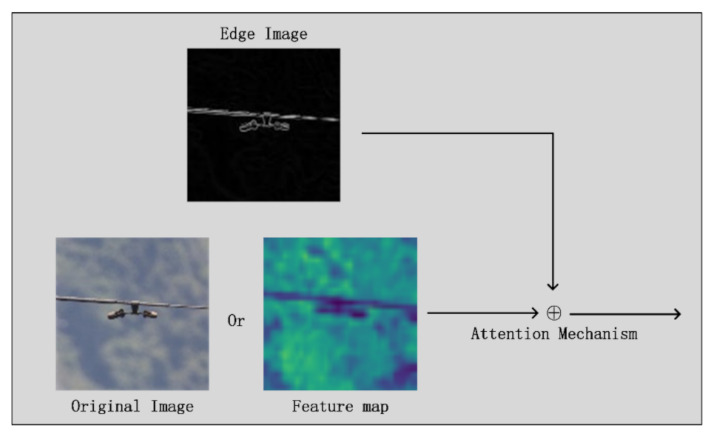
Schematic diagram of the attention mechanism.

After obtaining the edge information in the image using the canny algorithm, it can be used to produce positive effects. The attention mechanism [42] originated in the field of NLP and has been introduced into computer vision in recent years. As shown in Figure 2, by introducing additional convolution operations, the attention mechanism can focus on the additional information being added.

The attention mechanism is based on the edge information obtained by the canny algorithm, and performs a convolution operation to obtain the attention weight matrix a. The expression of the convolution operation is shown in Equation (13).

(13) $$I_{A}^{i}=\mathrm{Softmax}(I^{i}W_{A}^{i}+b_{A}^{i}),\mathrm{for}\;i=1,2$$

where $I^{i}$ represents the input image, ${\left\{W_{A}^{i},b_{A}^{i}\right\}}_{i=1}^{2}$ represents the parameter of the convolution operation, and $\mathrm{Softmax}\left(\cdot \right)$ represents the SoftMax function used for normalization.

We multiplied the resulting attention weight matrix with the corresponding input image to obtain the final output:

(14) $$I_{A}=(I_{A}^{1}⊗I^{1})⊕(I_{A}^{2}⊗I^{2})$$

where $I_{A}$ represents the final output result of the attention mechanism, $I^{1}$ and $I^{2}$ represent the input images, and the symbols ⊗ and ⊕ represent the multiplication and addition elements of the matrix.

Attention mechanism is used in ResNet101 to send the edge image output by the canny algorithm to the network to enhance the network’s ability to focus on the ground truth region during feature extraction. We used an attention mechanism in layers 1, 2, and 3 of ResNet because the network focuses on the low-level features of the input image in the early stage of feature extraction. At the fourth and fifth layers, the output is a feature map with highly abstract semantics. At this time, the introduction of the attention mechanism containing the edge map interferes with the effect of the feature map. A follow-up sensitivity analysis on where the attention mechanism is introduced proves our point.

### 4.3. Feature Fusion Network

After introducing edge detection and attention mechanisms, our framework improved to a certain extent. However, in the inspection data of overhead transmission lines captured by UAVs, the vibration damper is a small target object. When ResNet101 performs feature extraction, the deep network responds easily to semantic features and the shallow network responds easily to image features. This feature leads to a problem: although the high-level network can respond to semantic features, due to the small size of the Feature Map it does not contain much geometric information, which is not conducive to object detection. This problem is more pronounced for small-sized object detection. The vibration damper easily disappears in the feature map output by the fifth layer of ResNet because the target is small.

The disappearance of the vibration damper feature leads to a decrease in detection accuracy.

It is natural to think that a feature map that combines deep and shallow features can be used to meet the needs of small target detection. FPN [43] is a network structure that adopts this idea. FPN uses the idea of image pyramid to solve the problem of difficulty in detecting small-sized objects in object detection scenes. The traditional image pyramid method uses a multiscale image input to construct multiscale features. The biggest problem with this approach is that the recognition time is *k* times the recognition time of a single image, where k is the number of scaled dimensions.

To improve the detection speed, methods such as Faster R-CNN [46] use a single-scale Feature Map, but the single-scale feature map limits the detection capability of the model, especially for samples with extremely low coverage in the training set (such as larger and smaller samples). Unlike Faster R-CNN, which only uses the top-level Feature Map, SSD [47] uses the hierarchical structure of convolutional networks, starting from conv4_3 of VGG [48], and obtains multiscale Feature Maps through different network layers. Although this method can improve accuracy and does not increase the test time, while it does not use the low-level Feature Map, these low-level features are very helpful for detecting small objects. In response to the above problems, FPN adopts the form of a Feature Map in the pyramid of SSD.

**Figure 3:**
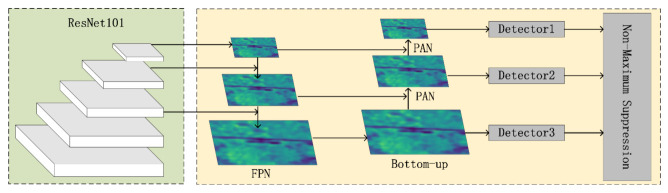
The Feature Fusion Network used for feature transfer containing two parts: the FPN and the Bottom-up module.

Different from SSD, FPN not only uses deep Feature Map in VGG, but also applies shallow Feature Map. These Feature Maps are efficiently integrated through bottom-up, top-down, and lateral connections, which improve the accuracy without greatly increasing the detection time. Therefore, as shown in Figure 3, this article refers to these practices and introduce a structure composed of FPN and bottom-up after the third, fourth, and fifth layers of ResNet101 so that the semantics and lines of the final output feature maps of the three scales’ layer features are more abundant.

**Figure 4:**
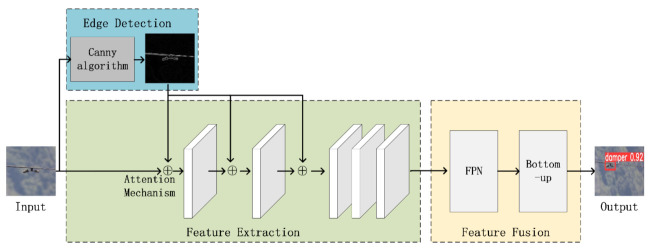
The realization of detection of vibration dampers is divided into three parts: Edge Detection, Feature Extraction, Feature Fusion. First, Edge Detection is used to provide edge information. then Feature Extraction and Feature Fusion are used to obtain feature maps for vibration dampers. Finally, the detection results can be obtained from classifier of YOLOv4.

DamperYOLO was trained after all framework components were introduced. The training process is as described in Algorithm 1. As shown in Figure 4, the Edge Detection module, the ResnNet101 backbone, Attention Mechanism, the FPN and Bottom-up framework are used to construct the entire vibration damper detection process.


**Algorithm 1: The Training Process of DamperYOLO.**
Input: Original damper image set $I=\left\{I_{1},\cdot \cdot \cdot ,I_{N}\right\}$ that each image contains dampers.Output: DamperYOLO after training.1: Initialize DamperYOLO with random weights;2: repeat3: for i in 1~epochs do 4:   for j in 1~N do 5:    Image augment for $I_{j}$; 6:   Extract feature map using ResNet101; 7:   Output detection results using YOLO;8:   Calculate the penalty value via Formula (2), (5) and (6);9:   Minimize Formula (1) to update the parameters of DamperYOLO; 10:  end for11: end for12: until DamperYOLO completes convergence13: return

## 5. Experiments and Analysis

### 5.1. Experiment Description

#### 5.1.1. Dataset

A dataset of vibration dampers for overhead transmission lines is required for the proposed theoretical validation and experimental analysis. At present, although there is a lot of research work on vibration dampers, but there is no completely public vibration damper detection dataset. Moreover, most of the vibration damper data in the article were obtained by geometric transformation methods such as flipping, cutting, and scaling. An insufficient number of vibration dampers would make it difficult to verify the correctness of the proposed theory. Therefore, a dataset was made for vibration damper detection based on the real UAV cruise video of overhead transmission lines, and named DamperDetSet. In the process of making the DamperDetSet dataset, LabelMe was used as a data labeling tool to label the positions of all existing line vibration dampers in the original image. The callout box was kept as close as possible to the smallest enclosing rectangle of the target area.

DamperDetSet contains a total of 3000 images, each of which contains vibration dampers, and the types of vibration dampers are not unique, such as hippocampus antislip vibration dampers, hook wire vibration dampers, etc. We randomly divided all 3000 images into a training set and a test set. The training set contains 2500 images and the test set contains 500 images. The ratio of training set and test set is 5:1. In addition, as the dataset is obtained by shooting with UAVs, the presentation angle of the vibration damper in the image is not unique, which also puts forward higher requirements for the robustness of the model.

#### 5.1.2. Experiment Configuration

In terms of hyperparameter settings in the experiment, we trained DamperYOLO for a total of 200 epochs. The learning rate of the first 100 epochs remained unchanged, and the learning rate of the last 100 epochs gradually decreased to 0. In terms of experimental software settings, all our programs were written in Python language and integrated based on the Pytorch 1.4 platform. In the system environment of the experimental platform, Ubuntu18.04 was used as the operating system. In terms of the hardware environment of the experimental platform, an NVIDIA RTX 2080 GPU was used as the main equipment for training calculation, matched with an AMD R5-3600X CPU and 32 GB RAM.

### 5.2. The Baselines

In the following experiments, we chose one-stage, two-stage, and anchor-free methods as comparison methods.

YOLOv4 [37]: This method is the latest achievement of the YOLO series. After continuing the advantages of the previous work, it introduces the structure of FPN + PAN, which improves the transferability of features in the network; it is also the basis of our proposed model.

Cascade R-CNN [49]: This framework is the latest achievement of the R-CNN series. It creatively introduces a cascade structure. The detection accuracy is state-of-the-art, but its excellent performance consumes a lot of computational resources.

CenterNet [50]: This method is a heatmap-based detection method rather than anchor-based, which has the advantage of fast testing and low space occupancy.

SSD [47]: SSD is another classic one-stage object detection method. It initially utilizes multiple detectors.

RetinaNet [51]: RetinaNet is based on FPN [43], and its contribution is to propose focal loss to solve the problem of category imbalance.

### 5.3. Qualitative Evaluation

**Figure 5:**
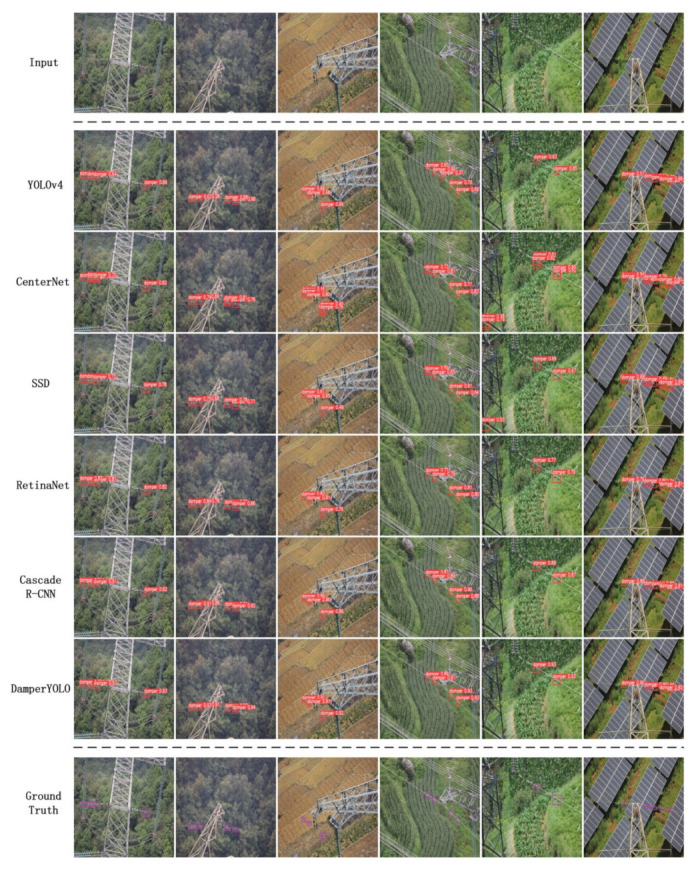
Test examples of each model on the DamperDetSet dataset. Experimental results show that the performance of DamperYOLO is similar to Cascade R-CNN, better than SSD, RetinaNet and YOLOv4 in one-stage class, and CenterNet.

To visually compare the difference between the detection effect of DamperYOLO and other baselines, we conducted qualitative analysis and comparison experiments based on the DamperDetSet dataset. The experimental results are shown in Figure 5. As can be seen from Figure 5, under the same test image, the detection effect of CenterNet is not stable enough. This proves that calculation of the heat map will be greatly disturbed by the current anchor-free algorithm in the face of complex scenes such as transmission lines. The performance of the two-stage Cascade R-CNN is very superior. As the latest framework of the R-CNNs series, the results obtained by the second iteration based on the proposal are more accurate. There is also room for improvement in the performance of a single-level SSD. And using VGG16 as a backbone may be weaker than the ResNet-like feature extraction network in feature extraction. RetinaNet and YOLOv4 perform better. Both of them benefit from the latest research results in one-stage. They can obtain high performance with only one calculation, but the edge detection effect of vibration damper still needs to be improved. Finally, DamperYOLO outperforms other one-stages. The detection result image of DamperYOLO proves that our proposed improvement strategy is effective, and its performance is no less than that of Cascade R-CNN.

### 5.4. Quantitative Evaluation

We compared other baselines and performed the quantitative analysis shown in Table 2 with AP in the COCO [52] dataset as the evaluation standard. The calculation of AP is based on the ground truth and the IOU of the prediction result. The calculation formula is shown in Equation (3). The AP calculation results were selected when the IOU was 0.5, 0.7, and 0.9 as the evaluation basis, so that the performance difference of the model under different pressure levels could be more comprehensively evaluated.

As can be seen from Table 2, under the same test picture, thanks to the two-stage detection strategy, the performance of Cascade R-CNN was still stable, and its performance under various AP standards was at the forefront; however, its good score came at the cost of great computation time.

The one-stage RetinaNet and YOLOv4 performed similarly, and YOLOv4 slightly outperformed RetinaNet. Compared with SSD, both of them had a certain degree of lead in terms of indicators, and the latest training tricks available from analysis confers an advantage in accuracy. In addition, the calculation speed of these three methods is faster than Cascade R-CNN, without the intermediate step of proposal, which shortens the calculation time considerably.

The anchor-free based CenterNet had the lowest score; so, it can be concluded that the calculation of the heatmap is very susceptible to interference from similar objects in the background. However, the advantage of the anchor-free class method is that the calculation speed is much faster than other baselines, which is a huge advantage for scenarios with extremely high real-time requirements.

Our proposed DamperYOLO takes the lead on AP, but the score of YOLOv4 is lower than Cascade R-CNN; therefore, the edge extraction, attention mechanism, and feature fusion structure proposed in this paper are better than Cascade R-CNN. The calculation speed of DamperYOLO was similar to other one-stage class methods. Therefore, DamperYOLO is a model with a balance between speed and accuracy.

### 5.5. Sensitivity Analysis

In this section, multiple sets of sensitivity analysis are performed on each component of DamperYOLO, which includes the choice of backbone, edge extraction, the attention mechanism, the number of training iterations, and the minimum amount of training data.

#### 5.5.1. Backbone

We conducted a sensitivity analysis on the backbone used by DamperYOLO while retaining other improvements. As shown in Table 3, the CSPDarknet53 used by YOLOv4 was improved based on ResNet50, so it performed better. In addition, the only objects need to be detected were dampers. Therefore, we believe that it may be more effective to expand the number of network layers and improve the feature abstraction ability of the backbone. The performance of ResNet101 also supports our idea, but if network layers such as using ResNet152 are added, the improvement is limited, so ResNet101 is used as the backbone.

#### 5.5.2. Edge Extraction

To verify the effectiveness of preprocessing, a sensitivity analysis was performed on the image denoising, and edge detection used while retaining other improvements constant. Table 4 shows that, compared with not using any preprocessing strategy, using image denoising and edge extraction alone leads to a certain improvement in detection effect. If both are used, the AP50 increase by about five percentage points, which shows that the image augmentation method in this paper is effective.

#### 5.5.3. Attention Mechanism

The attention mechanism is an important mechanism pioneered in the field of NLP, and was developed in object detection in recent years. In order to verify the effect of adding an attention mechanism in different layers of ResNet101, we conducted a sensitivity analysis for the number of times an attention mechanism is introduced while retaining the other conditions. As shown in Table 5, when the attention mechanism was added to the first three layers of ResNet101, the detection effect improved to a certain extent. However, continuing to introduce attention-blocks containing edge information to the 4th and 5th layers caused a drop in detection accuracy. This is because there is more abstract information in the feature maps extracted by the fourth and fifth layers in ResNet101, and the edge information is the basic feature information. This is counterproductive and reduces the detection performance.

#### 5.5.4. Number of Epochs

The number of epochs for experimental training affects the performance of the model. Because the number of training epochs is not enough, the model is under-fitted, and the model has not yet fully learned to identify all the objects to be detected. Excessive training epochs reduce the robustness of the model, the parameters are limited by the existing training data, and the realization of unfamiliar data in the test set is reduced. Therefore, we conducted an evaluation test of the number of training times for the performance of the model, and the test results are shown in Table 6. It can be seen from the table that when the training epoch is 200, the model is the most balanced.

#### 5.5.5. Minimum Training Data Experiment

Changes in the amount of training data also affect the final performance of the model. At the same time, by comparing the detection accuracy of the model with different amounts of data, the feature extraction ability of the model can be judged. As shown in Table 7, we conducted experiments with the minimum amount of data. From the results, it can be seen that when the amount of data decreases, the performance of the model has weak performance, which indicates that our data is sufficient. The model performance did not drop significantly until the test set dropped to 1750. Moreover, DamperYOLO had strong robustness and could still learn key feature information on small-scale datasets, which overcame the shortcomings of the previous model’s poor generalization ability to a certain extent.

### 5.6. Ablation Analysis

To analyze the functions of the different components of DamperYOLO, an ablative analysis was performed on DamperDetSet. As shown in Table 8, Model B had better indicators than Model A, which indicates that using ResNet101 as the backbone can better extract image features. Model C uses image augmentation for preprocessing, which improves the quality of the input image and provides the model with better training data. Compared with other stages, the performance of Model D has the highest improvement in detection effect. This indicates that the attention mechanism plays a sufficient role, because the attention mechanism allows the model to focus on the edge information of the damper when converging, with the help of the image enhancement model. In addition, it can be seen from other comparative experiments that the additional overhead brought by it is very low, so it is necessary for our task to add an attention mechanism to the backbone.

### 5.7. Computational Complexity

The network parameters and training time were recorded to evaluate the space and time complexity of the networks. As shown in Table 9, compared with Cascade R-CNN, DamperYOLO has a similar detection effect, but its parameters and training time are greatly reduced. Compared with YOLOv4, the space complexity and the training time are basically unchanged, because we only changed the backbone and added the attention mechanism on its basis, but a higher detection effect was achieved. In addition, CenterNet still consumes the least resources. The computational complexity of SSD is slightly higher than that of RetinaNet, but the detection effect is slightly worse.

## 6. Conclusions

We propose a power line vibration damper detection model named DamperYOLO based on a deep neural network that can detect the position of the vibration damper in drone inspection aerial images. DamperYOLO first uses the Canny algorithm to obtain the edge information of the original image, then uses the attention mechanism to introduce edge information into ResNet101 to guide feature extraction. Finally, it outputs a feature map that is more conducive to small target detection with the FPN structure. The following conclusions can be drawn through qualitative and quantitative experiments on the power line vibration damper detection dataset built in this paper. Compared with the current baselines in the object detection field, the DamperYOLO proposed in this paper can output state-of-the-art detection accuracy. The results of sensitivity analysis experiments show that edge detection, attention mechanism, and feature pyramid network all significantly improve the detection accuracy. The ablation analysis shows that the attention mechanism and the feature pyramid network improve the accuracy of the output detection results. In addition, DamperYOLO consumes space similar to the computational resources and baselines of other one-stage classes, but the detection accuracy can reach the level of Cascade R-CNN, which shows the superiority of our model. In the future, we will continue to introduce appropriate training tricks for the detection accuracy of DamperYOLO and explore the application of the model to other power line components.

## Figures and Tables

**Table 1:** Applied kernels of ResNet101 in DamperYOLO.

Layer	Output Size	Kernel Size
conv1	304 × 304	7 × 7, 64
conv2_x	152 × 152	$\left[\begin{array}{c} 1\times 1,64\\ 3\times 3,64\\ 1\times 1,\;256 \end{array}\right]$× 3
conv3_x	76 × 76	$\left[\begin{array}{c} 1\times 1,128\\ 3\times 3,128\\ 1\times 1,512 \end{array}\right]$× 4
conv4_x	38 × 38	$\left[\begin{array}{c} 1\times 1,256\\ 3\times 3,256\\ 1\times 1,1024 \end{array}\right]$× 23
conv5_x	19 × 19	$\left[\begin{array}{c} 1\times 1,512\\ 3\times 3,512\\ 1\times 1,2048 \end{array}\right]$× 3

**Table 2:** APs of the different models.

Model	DamperDetSet			FPS
	AP_50_	AP_70_	AP_90_	
YOLOv4	88.23	80.67	73.26	71
SSD	85.71	78.34	71.38	70
RetinaNet	87.18	79.62	72.70	73
CenterNet	84.38	77.25	69.42	118
Cascade R-CNN	92.26	89.52	81.43	31
DamperYOLO	92.62	89.67	81.24	74

**Table 3:** APs of different backbones.

Backbone	DamperDetSet			FPS
	AP_50_	AP_70_	AP_90_	
CSPDarknet53	88.20	80.58	73.28	72
VGG16	82.18	76.54	67.91	71
ResNet50	84.12	77.62	70.42	78
ResNet101(ours)	92.62	89.67	81.24	74
ResNet152	93.25	89.97	82.16	68

**Table 4:** APs of different preprocessing methods.

Preprocessing Method	DamperDetSet			FPS
	AP_50_	AP_70_	AP_90_	
No preprocessing	87.18	79.52	71.83	79
Image denoising	88.92	81.93	73.65	78
Edge extraction	91.25	86.74	79.17	77
Image denoising + Edge extraction	92.62	89.67	81.24	74

**Table 5:** APs of different introduction times of the attention mechanism.

Introduced Layer	DamperDetSet			FPS
	AP_50_	AP_70_	AP_90_	
None	86.28	77.36	70.03	81
C1	87.83	80.23	71.37	80
C1, C2	91.38	84.61	77.42	77
C1, C2, C3	92.62	89.67	81.24	74
C1, C2, C3, C4	93.14	90.15	81.92	74
C1, C2, C3, C4, C5	89.27	83.32	73.52	73

**Table 6:** APs of different epoch numbers.

Number of Epochs	DamperDetSet			FPS
	AP_50_	AP_70_	AP_90_	
50	71.63	60.62	41.37	79
100	80.51	72.27	65.23	77
150	84.15	80.16	74.38	75
200	92.62	89.67	81.24	74
250	93.31	88.65	80.47	74

**Table 7:** Results of minimum training data experimental.

The Amount of Training Set	DamperDetSet			FPS
	AP_50_	AP_70_	AP_90_	
2500 (100%)	92.62	89.67	81.24	74
2250 (90%)	89.51	86.28	78.83	75
2000 (80%)	85.39	81.75	75.41	74
1750 (70%)	82.41	77.40	71.68	74
1500 (60%)	73.97	69.62	64.01	72

**Table 8:** The results of the ablation analysis.

Model	Architecture	AP_50_	AP_70_	AP_90_
A	YOLOv4	86.21	78.45	70.96
B	A + ResNet101	88.57	82.36	73.72
C	B + Edge Extraction	90.82	84.24	76.50
D	C + Attention Mechanism	92.62	89.67	81.24

**Table 9:** Network parameters (Param.) and training time of the different models.

Model	Param.	Training Time (h)
YOLOv4	28 M	6.38
SSD	34 M	7.46
RetinaNet	32 M	7.03
CenterNet	14 M	4.05
Cascade R-CNN	184 M	49.84
DamperYOLO	30 M	6.92

## Data Availability

The data in this paper are undisclosed due to the confidentiality requirements of the data supplier.

## References

[1] Bao W., Ren Y., Wang N., Hu G., Yang X. (2021). Detection of Abnormal Vibration Dampers on Transmission Lines in UAV Remote Sensing Images with PMA-YOLO. Remote Sens..

[2] Wu H., Xi Y., Fang W., Sun X., Jiang L. Damper detection in helicopter inspection of power transmission line. Proceedings of the 2014 4th International Conference on Instrumentation and Measurement, Computer, Communication and Control (IMCCC).

[3] Ma Y., Li Q., Chu L., Zhou Y., Xu C. (2021). Real-Time Detection and Spatial Localization of Insulators for UAV Inspection Based on Binocular Stereo Vision. Remote Sens..

[4] Hinas A., Roberts J.M., Gonzalez F. (2017). Vision-Based Target Finding and Inspection of a Ground Target Using a Multirotor UAV System. Sensors.

[5] Huang S., Han W., Chen H., Li G., Tang J. (2021). Recognizing Zucchinis Intercropped with Sunflowers in UAV Visible Images Using an Improved Method Based on OCRNet. Remote Sens..

[6] Popescu D., Stoican F., Stamatescu G., Chenaru O., Ichim L. (2019). A Survey of Collaborative UAV–WSN Systems for Efficient Monitoring. Sensors.

[7] Zhang Y., Yuan X., Li W., Chen S. (2017). Automatic Power Line Inspection Using UAV Images. Remote Sens..

[8] Liu Y., Li J.X., Xu W., Liu M.Y. A method on recognizing transmission line structure based on multi-level perception. Proceedings of the International Conference on Image and Graphics.

[9] Huang X., Zhang X., Zhang Y., Zhao L. (2020). A method of identifying rust status of dampers based on image processing. IEEE Trans. Instrum. Meas..

[10] Jin L.J., Yan S.J., Liu Y. (2012). Vibration damper recognition based on Haar-Like features and cascade adaboost classifier. J. Syst. Simul..

[11] Zhang K., Hou Q., Huang W. Defect Detection of Anti-vibration Hammer Based on Improved Faster R-CNN. Proceedings of the 2020 7th International Forum on Electrical Engineering and Automation (IFEEA).

[12] Bao W., Ren Y., Liang D., Yang X., Xu Q. Defect Detection Algorithm of Anti-vibration Hammer Based on Improved Cascade R-CNN. Proceedings of the 2020 International Conference on Intelligent Computing and Human-Computer Interaction (ICHCI).

[13] Hickey C., Young P., Mayomi T., Noctor J. Fault Investigation and Analysis of an Overhead Transmission Line Vibration Damper. Proceedings of the 2021 56th International Universities Power Engineering Conference (UPEC).

[14] Xiao S., Wang H., Ling L. Research on a novel maintenance robot for power transmission lines. Proceedings of the 2016 4th International Conference on Applied Robotics for the Power Industry (CARPI).

[15] Song W., Zuo D., Deng B., Zhang H., Xue K., Hu H. (2016). Corrosion defect detection of earthquake hammer for high voltage transmission line. Chin. J. Sci. Instrum..

[16] Yang H., Guo T., Shen P., Chen F., Wang W., Liu X. Anti-vibration hammer detection in UAV image. Proceedings of the 2017 2nd International Conference on Power and Renewable Energy (ICPRE).

[17] Liu Y., Wen S., Chen Z., Zhang D. Research of the Anti-vibration Hammer Resetting Robot Based on Machine Vision. Proceedings of the 2020 Chinese Control and Decision Conference (CCDC).

[18] Chen X., Wu Y., Zhao L. (2010). Identification of OPGW vibration damper based on random Hough transformation. Heilongjiang Dianli Jishu Heilongjiang Electric. Power.

[19] Miao S., Sun W., Zhang H. (2010). Intelligent visual method based on wavelet moments for obstacle recognition of high voltage transmission line deicer robot. Jiqiren.

[20] Pan L., Xiao X. Image recognition for on-line vibration monitoring system of transmission line. Proceedings of the 2009 9th International Conference on Electronic Measurement & Instruments.

[21] Liu Y., Jin L. Vibration Damper Recognition of Transmission System Based on Unmanned Aerial Vehicles. Proceedings of the 2011 Asia-Pacific Power and Energy Engineering Conference.

[22] Guo J., Xie J., Yuan J., Jiang Y., Lu S. Fault Identification of Transmission Line Shockproof Hammer Based on Improved YOLO V4. Proceedings of the 2021 International Conference on Intelligent Computing, Automation and Applications (ICAA).

[23] Wang W., Wang Z., Liu B., Yang Y., Sun X. Typical Defect Detection Technology of Transmission Line Based on Deep Learning. Proceedings of the 2019 Chinese Automation Congress (CAC).

[24] Zhang Z., Jiang W., Yang J. An Improved Quantization Algorithm for Electric Power Inspection. Proceedings of the 2021 9th International Electrical Engineering Congress (iEECON).

[25] Liu C., Wu Y., Liu J., Sun Z. (2021). Improved YOLOv3 Network for Insulator Detection in Aerial Images with Diverse Background Interference. Electronics.

[26] Sadykova D., Pernebayeva D., Bagheri M., James A. (2020). IN-YOLO: Real-Time Detection of Outdoor High Voltage Insulators Using UAV Imaging. IEEE Trans. Power Deliv..

[27] Zhao Z., Zhen Z., Zhang L., Qi Y., Kong Y., Zhang K. (2019). Insulator Detection Method in Inspection Image Based on Improved Faster R-CNN. Energies.

[28] Chen W., Li Y., Li C. (2020). A Visual Detection Method for Foreign Objects in Power Lines Based on Mask R-CNN. Int. J. Ambient. Comput. Intell. IJACI.

[29] Kang G., Gao S., Yu L., Zhang D. (2019). Deep Architecture for High-Speed Railway Insulator Surface Defect Detection: Denoising Autoencoder with Multitask Learning. IEEE Trans. Instrum. Meas..

[30] Cheng L., Liao R., Yang L., Zhang F. (2018). An Optimized Infrared Detection Strategy for Defective Composite Insulators According to the Law of Heat Flux Propagation Considering the Environmental Factors. IEEE Access.

[31] Yu C., Pan W., Lei X., Yu G., Qin W., Zhu K., Zheng H. Simulation of electric field and potential transfer arc during the on-line process of the live working anti-vibration hammer robot. Proceedings of the 2021 International Conference on Electrical Materials and Power Equipment (ICEMPE).

[32] Diana G., Falco M., Cigada A., Manenti A. (2000). On the measurement of overhead transmission lines conductor self-damping. IEEE Trans. Power Deliv..

[33] Si J., Rui X., Liu B., Zhou L., Liu S. (2019). Study on a New Combined Anti-Galloping Device for UHV Overhead Transmission Lines. IEEE Trans. Power Deliv..

[34] Redmon J., Divvala S., Girshick R., Farhadi A. You only look once: Unified, real-time object detection. Proceedings of the 2016 IEEE Conference on Computer Vision and Pattern Recognition (CVPR).

[35] Redmon J., Farhadi A. YOLO9000: Better, faster, stronger. Proceedings of the IEEE Conference on Computer Vision and Pattern Recognition (CVPR).

[36] Redmon J., Farhadi A. (2018). YOLOv3: An Incremental Improvement. arXiv.

[37] Bochkovskiy A., Wang C.Y., Liao H. (2020). YOLOv4: Optimal Speed and Accuracy of Object Detection. arXiv.

[38] Wang C.Y., Liao H.M., Wu Y.H., Chen P.Y., Hsieh J.W., Yeh I.H. CSPNet: A new backbone that can enhance learning capability of cnn. Proceedings of the IEEE Conference on Computer Vision and Pattern Recognition Workshop (CVPR Workshop).

[39] Zheng Z., Wang P., Liu W., Li J., Ye R., Ren D. Distance-IoU Loss: Faster and better learning for bounding box regression. Proceedings of the AAAI Conference on Artificial Intelligence (AAAI).

[40] He K., Zhang X., Ren S., Sun J. Deep Residual Learning for Image Recognition. Proceedings of the 2016 IEEE Conference on Computer Vision and Pattern Recognition (CVPR).

[41] Canny J. (1986). A Computational Approach to Edge Detection. IEEE Trans. Pattern Anal. Mach. Intell..

[42] Bahdanau D.B., Kyunghyun C., Yoshua B. (2014). Neural Machine Translation by Jointly Learning to Align and Translate. arXiv.

[43] Lin T.Y., Dollár P., Girshick R., He K., Hariharan B., Belongie S. Feature pyramid networks for object detection. Proceedings of the IEEE Conference on Computer Vision and Pattern Recognition (CVPR).

[44] Tao C., Ma K.K., Chen L.H. (1999). Tri-state median filter for image denoising. IEEE Trans. Image Process..

[45] Zhang X.M., Xu B.S., Dong S.Y., Gan X.M. (2004). Adaptive median-weighted mean hybrid filter. Opt. Tech..

[46] Ren S., He K., Girshick R.B., Sun J. (2017). Faster R-CNN: Towards Real-Time Object Detection with Region Proposal Networks. IEEE Trans. Pattern Anal. Mach. Intell..

[47] Liu W., Anguelov D., Erhan D., Szegedy C., Reed S., Fu C., Berg A.C. SSD: Single shot multibox detector. Proceedings of the European Conference on Computer Vision (ECCV).

[48] Simonyan K., Zisserman A. (2014). Very Deep Convolutional Networks for Large-Scale Image Recognition. arXiv.

[49] Cai Z., Vasconcelos N. Cascade R-CNN: Delving into High Quality Object Detection. Proceedings of the IEEE Conference on Computer Vision and Pattern Recognition.

[50] Zhou X., Wang D., Krähenbühl P. (2019). Objects as Points. arXiv.

[51] Lin T.Y., Goyal P., Girshick R., He K., Dollár P. (2020). Focal loss for dense object detection. IEEE Trans. Pattern Anal. Mach. Intell..

[52] Lin T.Y., Maire M., Belongie S., Hays J., Zitnick C.L. (2014). Microsoft COCO: Common Objects in Context. Proceedings of the European Conference on Computer Vision-ECCV 2014, Lecture Notes in Computer Science.

